# Prevalence of depression among people living with HIV in rural hospitals in South-Western Nigeria-Association with clinico-demographic factors

**DOI:** 10.1186/s12981-023-00586-0

**Published:** 2023-12-16

**Authors:** Waheed Adeola Adedeji, Qing Ma, Abiodun Muhammed Raji, Raymond Cha, Olaniran Mudasiru Rasaki, Alan Hutson, Babafemi O. Taiwo, Man E. Charurat, Oyindamola B. Yusuf, Fatai Adewale Fehintola, Oye Gureje, Gene D. Morse

**Affiliations:** 1 Department of Pharmacology and Therapeutics, College of Medicine, University of Ibadan, Ibadan, Nigeria; 2 Department of Clinical Pharmacology, University College Hospital, Ibadan, Nigeria; 3 Center for Integrated Global Biomedical Sciences, School of Pharmacy and Pharmaceutical Sciences, University at Buffalo, Buffalo, NY USA; 4 Department of Medical Microbiology and Parasitology, University College Hospital, Ibadan, Nigeria; 5 Department of Clinical Pharmacy, Faculty of Pharmacy, University of Ibadan, Ibadan, Nigeria; 6 Department of Biostatistics and Bioinformatics, Roswell Park Comprehensive Cancer Center, Buffalo, NY USA; 7 Division of Infectious Diseases, Northwestern University Feinberg School of Medicine, Chicago, IL USA; 8 Center for International Health, Education, and Biosecurity, Institute of Human Virology, University of Maryland School of Medicine, Baltimore, MD USA; 9 Department of Epidemiology and Medical Statistics, University of Ibadan, Ibadan, Nigeria; 10 Department of Psychiatry, University of Ibadan, Ibadan, Nigeria

**Keywords:** HIV/AIDS, PLWH, ART, Depression, Mental disorder, Nigeria

## Abstract

Major depression is the most common neuropsychiatric disorder among people living with HIV (PLWH) and is predictive of high morbidity and mortality among them. This study estimated the prevalence and explored factors associated with depression among PLWH in two rural secondary health facilities providing anti-retroviral therapy (ART) services in Southwestern Nigeria between September and December 2020. The Patient Health Questionnaire-9 (PHQ-9) was used to screen and identify PLWH aged 18 years or older with depression. Descriptive statistics, bivariate and multivariate analyses were performed with SPSS version 23. A total of 172 respondents were screened. The prevalence of depression was 16.3% (95% CI 11.1%, 22.7%). Mild, moderate, and moderately severe depression was identified in 17 (9.9%), 8(4.7%) and 3(1.7%) of the participants, respectively. One (0.6%) respondent had suicidal ideation. Of PLWH with any depression, 20/28(71.4%) were within the 40–59 years of age range. None of the participants was on antidepressants. The factor most associated with depression was hypertension, with adjusted odd ratios of 9.8(95% CI 3.5–27.3, p < 0.0001). The study highlights the importance of screening for the severity of depression among PLWH in rural hospitals providing ART services in Africa. PLWH with comorbid hypertension were more likely to suffer from some form of depression.

## Introduction

Globally, there has been a positive change in the demographics of people living with HIV (PLWH) in the last three decades due to the availability and effectiveness of anti-retroviral therapy (ART) [1]. PLWH are faced with a paradigm change from a disease with high morbidity and mortality, mainly from infectious diseases to a more significant burden of non-communicable diseases [2, 3].

Depression is the most common neuropsychiatric disorder among PLWH. Depression results in poor physical health [4], reduced ART adherence [5–7], treatment failure [8], reduced quality of life [9, 10], increased suicidal behaviours [11, 12] and HIV-related mortality among PLWH [13].

The risk of depression is higher among PLWH when compared with the general population. For example, a study in the United States (US) reported a prevalence of 58% elevated depressive scores among PLWH compared to 33% among HIV seronegative individuals [14].

Globally, the prevalence of depression among PLWH ranges from 28 to 34%, with a higher prevalence in low- and middle-income countries in contrast to developed countries [15]. The prevalence of depression among PLWH is 22 to 32% in the US [16, 17], 43.0% in China [18], 29.3–47.5% in East Africa [19], 9 to 32% in sub-Saharan Africa [20], and 23–28% in Nigeria [21, 22]. However, despite the high burden of depression among PLWH, it remains a neglected public health problem in sub-Saharan Africa [20].

In Africa, there are disparities in the access to health care and availability of specialised services, including mental health care, in rural areas. It is unknown whether the prevalence of depression is similar between PLWH living in rural and urban areas in sub-Saharan Africa. This study determined the prevalence and explored factors associated with major depression among PLWH in two rural state hospitals providing ART services in Southwestern Nigeria.

## Methods

### Study design

This was a descriptive cross-sectional study.

### Setting

This study was conducted at the State Hospital Saki and General Hospital Okeho in the Oke-Ogun Region of Oyo State, Southwest Nigeria. Oke-Ogun is in the Oyo-North senatorial district, a rural community with poor socioeconomic indices. Most inhabitants are Yoruba; other ethnic groups like Igbo, Hausa, and Fulani constitute minority populations. Some foreigners like the Beninese and Togolese also reside in the region.

Secondary healthcare facilities are present in most of the region’s major towns, with six health facilities offering ART services. Both hospitals selected have similar characteristics, including the demographics of the PLWH receiving ART services in the hospitals.

### Participants

The target population comprises PLWH accessing HIV care and support services at the ART clinics. The inclusion criteria included consenting male or female PLWH aged 18 years and above, residents of the Okeogun area and accessing outpatient HIV care and support services at the selected ART clinics.

### Data collection

A semi-structured questionnaire was adapted from previous studies [22–25]. The interviewer-administered questionnaire included sections incorporating the sociodemographic characteristics, PHQ-9, and clinical information, which were retrieved from the participants’ case notes. Participants were screened for depression using PHQ-9. This tool has been validated and used in sub-Saharan Africa [25–27]. The final PHQ-9 score was graded to determine the presence (PHQ9 ≥ 5) or absence of a depression (PHQ9 < 5) and its severity as mild depression (5–9), moderate depression (10–14), moderately severe depression (15–19), and severe depression (20–27). The questionnaire was translated into the local language (Yoruba). Trained research assistants administered the questionnaires in local language under the supervision of the principal investigator. A consecutive visit-based sampling technique was used based on clinic attendance. The study was conducted between September and November 2020.

### Statistical analysis

The data obtained were cleaned and entered into SPSS version 23. The sociodemographic characteristics were summarised with descriptive statistics (frequency and proportions) and presented as text and table. The inter-rater reliability, Cohen’s Kappa was 0.69. The quantitative variables were summarised with a mean (standard deviation) or median (interquartile range) if not normally distributed. Chi-square test, odds ratios (ORs) and 95% confidence intervals (CIs) were used to characterise factors associated with depression among PLWH. Fisher’s exact test was used for small cell counts. Binary outcomes were examined in a univariate and multivariate fashion using logistic regression. The level of significance was set at 5%.

## Results

### Sociodemographic and clinical characteristics of the participants

**Fig. 1:**
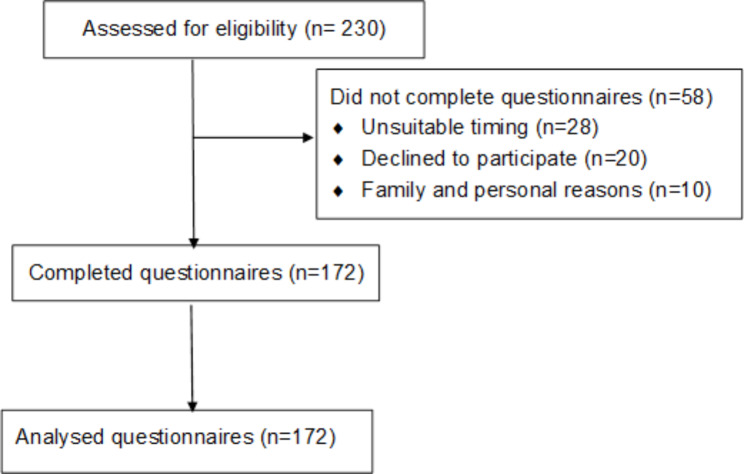
Flow diagram of recruitment of study participants

Of the 230 participants approached, 172 (75%) completed the questionnaires and were screened for depression (Fig. 1). There were no characteristic differences between those that refused participation and those included in the study. Of the participants, 94 (54.7%) were aged 40–59 years, 128 (74.4%) were females, and 138 (80.2%) were married. Table 1 shows other sociodemographic characteristics of the participants. The mean age (standard deviation) of the participants was 44.3 ± 11.7 years. The median (interquartile range) monthly income was 15,000 naira (15,000). The median (interquartile range) duration of ART was 4.5 (7.5 years), and the viral load was 39.8 (287.5) copies per millilitre. About three-quarters of the participants on ART had viral suppression (HIV viral loads < 200 copies per millilitres). The median (interquartile range) of the latest CD4 count was 520 (335) cells/mm^3^. The prevalence of hypertension among the participants was 14%, and only one (0.6%) participant had suicidal ideations, gestures, or attempts.

### Prevalence of major depression among PLWH

The prevalence of depression (95% CI) was 16.3% (11.1%, 22.7%) overall, with 17.2% (11.1%, 24.9%) in females and 13.6% (5.2%, 27.4%) in males. No statistically significant difference between the difference in the proportion of depression prevalence between males and females (OR 0.761; 95% CI 0.287–2.019, p = 0.582). Among the 28 participants with depressive disorders, 17 (9.9%) presented with mild depression, 8 (4.7%) had moderate depression, and 3 (1.7%) had moderately severe depression. Most of the participants with depression, 20/28 (71.4%), were within the 40–59 years age range.

### Factors associated with depression among PLWH

In the bivariate analysis (Table 2), the following factors were significantly associated with depression: hypertension (OR 10.5; 95% CI 4–27.5, p < 0.0001) and efavirenz use (OR 1.22; 95% CI 1.14–1.32, p = 0.042). However, after multivariate analysis, the only factor associated with depression among PLWH was hypertension (AOR 9.8; 95% CI 3.5–27.3, p < 0.0001).

**Table 1:** Sociodemographic characteristics of 172 PLWH in two rural secondary health facilities, Southwest, Nigeria, 2020

Variables	Frequency	Proportion
Age Group(years)		
Below 20	3	1.7
20–39	51	29.6
40–59	94	54.7
60 & above	24	14
Gender		
Male	44	25.6
Female	128	74.4
Marital status		
Single	6	3.5
Married	138	80.2
Widowed	17	9.9
Divorced/separated	11	6.4
Educational Level		
Nil	54	31.4
Primary	60	34.9
Secondary	45	26.1
Tertiary	13	7.6
Religion		
Islam	117	68.0
Christianity	55	32.0
Race		
Yoruba	163	94.7
Hausa/Fulani	5	3.0
Beninoise	4	2.3
Occupation		
Professional	15	8.7
Semi-skilled	97	56.4
Unskilled	50	29.1
Others*	10	5.8
Average Monthly Income (Naira)		
Less than 18,000	99	57.6
Greater than or equal to 18,000	73	42.4
Alcohol intake	12	7.0
Smoking	7	4.1
Substance use (Marijuana)	1	0.6

*Unemployed

**Table 2:** Factors associated with depression among 172 PLWH in two rural secondary health facilities, Southwest, Nigeria, 2020

Variables	No	Depression	Depression		OR (95% CI)	p-value
			N	%		
Age (years)						
<50	101	70.1	16	57.1	0.57 (0.25, 1.30)	0.177
≥50	43	29.9	12	42.9		
Sex						
Female	106	73.6	22	78.6	1.3 (0.5, 3.5)	0.582
Male	38	26.4	6	21.4		
Religion						
Islam	99	68.8	18	64.3	0.82 (0.35, 1.90)	0.643
Christianity	45	31.2	10	35.7		
Marital Status						
Married	112	80.2	26	92.9	3.7 (0.84, 16.5)	0.074
Not married	32	19.8	2	7.1		
Educational level						
Less than Secondary	94	65.3	20	71.4	1.3 (0.55, 3.23)	0.529
At least Secondary	50	34.7	8	28.6		
Hypertension						
Yes	11	7.6	13	46.4	10.5 (4.0, 27.5)	< 0.0001
No	133	92.4	15	53.6		
Diabetes mellitus						
No	142	98.6	28	100.0	0.84 (0.78, 0.89)	0.70
Yes	2	1.4	0	0.0		
Efavirenz use						
Yes	19	13.2	0	0.0	1.22 (1.14, 1.32)	0.046
No	125	86.8	28	100.0		
Viral suppression						
Yes	103	71.5	23	82.1	1.83 (0.65, 5.14)	0.246
No	41	28.5	5	17.9		
Alcohol intake						
Yes	8	5.6	4	5.6	0.35 (0.10, 1.27)	0.109
No	136	94.4	24	94.4		
Smoking						
No	139	96.5	26	92.9	0.5 (0.10, 2.54)	0.319
Yes	5	3.5	2	7.1		
Herbal concoction						
No	95	67.9	19	67.9	0.80 (0.72, 0.89)	0.847
Yes	49	32.1	9	32.1		
Duration on ART (years)						
<4.5	67	46.5	14	50.0	1.20 (0.51, 2.58)	0.736
≥4.5	77	53.5	14	50.0		
Average monthly income (Naira)						
<18,000	84	58.3	15	53.6	0.84 (0.37, 1.87)	0.641
≥18,000	60	41.7	13	46.4		

## Discussion

Depression is a non-communicable disease of global public health importance among PLWH [28]. This study determined the prevalence and explored factors associated with depression among PLWH in rural secondary health facilities providing ART services in Southwestern Nigeria. The prevalence of major depression among PLWH on ART was 16.3%. This finding is lower than that of other studies done in Africa in different places, Northern Tanzania, 20.9% [29], Ethiopia, 35.8% [30], Cameroon, 26.7% [31] and 30.4% in a recent systematic review and meta-analysis of non-communicable diseases burden among PLWH in sub-Saharan Africa [32]. However, our finding is similar to a systematic review and meta-analysis that reported the overall prevalence of major depression among PLWH in Sub-Saharan Africa using a diagnostic interview to be 15.3% [33]. The difference in the findings may also be due to variations in sample size, the study population (rural versus urban), the study period, the eligibility criteria and survey instruments used to assess depression. Most of these studies were performed in urban areas and tertiary hospitals, while our study was conducted in secondary health facilities in rural areas.

The odds of major depression were higher among those on efavirenz-containing ART in bivariate analysis. However, few participants were on efavirenz-containing ART, as the favoured first-line ART in Nigeria is dolutegravir-containing ART. Efavirenz has psychotropic properties, and chronic use has been associated with depression [34, 35]. Also, studies have reported improving depression after discontinuing long-term efavirenz treatment [36]. Contrarily, a systematic review in South Africa among PLWH treated with efavirenz reported depression as generally mild [37]. However, in this study, the association between efavirenz and major depression was not sustained after multivariate analysis.

Interestingly, hypertension is a chronic illness associated with depression, even among the general population [38]. In this study, those with hypertension had increased odds of having major depression. Kinyanda et al. in Uganda reported an association between depression and hypertension [39]. It has also been reported as one of the most prevalent NCDs among PLWH [40]. Screening and treating hypertension and other chronic non-communicable diseases among PLWH may reduce the burden of depression and improve their quality of life.

Female gender is a known risk factor for depression among PLWH [41–43]. However, in this study, gender was not statistically significantly associated with depression among PLWH. A probable reason for our findings is our study’s rural location, as women have more social support than in urban areas. Studies in sub-Saharan Africa also reported similar findings [44–46].

Unlike other studies, age, average monthly income, gender, religion, educational level, viral suppression, alcohol intake and ART duration were not statistically significantly associated with depression. However, the discrepancy may result from the small sample size of this study, the location of study participants (mainly rural dwellers) and the study period. This study was conducted during the COVID-19 pandemic, with a decline in clinic attendance by PLWH due to the lockdown by the government and fear of infection with COVID-19 by the people. Stigma, side effects, social support, treatment adherence, domestic abuse, and the number of children are other important covariates and confounders not included in the analysis and are significant limitations. Another limitation is recall bias, with a consequent effect on the study’s internal validity. Other limitations included inadequate sample size for multivariate analysis, the non-probability sampling method, and the hospital-based nature of the survey, which limited its generalisation.

## Conclusions

The study highlights the importance of depression screening among PLWH in rural hospitals providing ART services in Africa. PLWH with comorbid hypertension was associated with depression. There is a need to integrate mental health care into the ART services for PLWH in Sub-Saharan Africa.

## Data Availability

The data supporting this study’s findings are not openly available due to reasons of sensitivity and are available from the corresponding author upon reasonable request.
